# Research on the "multi-agent co-governance" system of unfair competition on internet platforms: Based on the perspective of evolutionary game

**DOI:** 10.1371/journal.pone.0301627

**Published:** 2024-04-18

**Authors:** Zhen Xu, Shudan Zheng

**Affiliations:** 1 School of Economics and Trade, Chongqing Business Vocational College, Chongqing, China; 2 School of Business Administration, Chongqing Vocational College of Light Industry, Chongqing, China; Sri Eshwar College of Engineering, INDIA

## Abstract

Unfair competition on internet platforms (UCIP) has become a critical issue restricting the platform economy’s healthy development. This paper applies evolutionary game theory to study how to utilize multiple subjects’ synergy to supervise UCIP effectively. First, the "multi-agent co-governance" mode of UCIP is constructed based on the traditional "unitary supervision" mode. Second, the government and internet platform evolutionary game models are built under two supervision modes. Finally, MATLAB is used to simulate and analyze the evolutionary stage and parameter sensitivity. In addition, we match the model’s evolutionary stage with China’s supervisory process. The results show that (1) the Chinese government’s supervision of UCIP is in the transitional stage from "campaign-style" to "normalization." (2) Moderate government supervision intensity is essential to guide the game system to evolve toward the ideal state. If the supervision intensity is too high, it will inhibit the enthusiasm for supervision. If the supervision intensity is too low, it cannot form an effective deterrent to the internet platforms. (3) When the participation of industry associations and platform users is low, it can only slow down the evolutionary speed of the game system’s convergence to the unfavorable state. Nevertheless, it cannot reverse the evolutionary result. (4) Maintaining the participation level of industry associations and platform users above a specific threshold value while increasing punishment intensity will promote the transition of government supervision from the "campaign-style" to the "normalization" stage. This paper provides ideas and references for the Chinese government to design a supervision mechanism for UCIP.

## Introduction

With the rise and iteration of emerging technologies, such as big data, cloud computing, and AI, the platform economy has shown a rapid development trend in recent years. It significantly improves resource allocation efficiency, smoothing the national economic cycle, and facilitates people’s lives. The platform economy is a vital engine driving macroeconomic growth [1]. The platform economy is an emerging economic form based on the internet platform as the primary carrier, data as the key production factor, the new generation of information technology as the core driving force, and network information infrastructure as the essential support. As the core subject of the platform economy, the number of internet platforms such as e-commerce, social networking, and live entertainment has grown exponentially, and their volume and market size are increasing yearly. For instance, users watch various entertainment and learning videos and share their daily lives on YouTube and TikTok platforms. Users purchase items for daily life, travel, food, and other items through Amazon, Shein, and Alibaba platforms, significantly improving shopping convenience. Users utilize the Google platform to search for issues, information, and news of interest. The emergence of internet platforms has profoundly changed people’s daily lives and dramatically improved the digital level of the economy and society. Simultaneously, various forms of unfair competition on internet platforms (UCIP) are also gradually emerging [2–4]. These practices seriously infringe on platform users’ rights and interests and restrict the platform economy’s healthy and orderly development. UCIP refers to internet platforms utilizing their market, technology, and other advantages to take actions that disrupt market order competition and harm the legitimate interests of competitors and users. These actions include "disclosure of user privacy, price discrimination, exclusive monopoly, self-preferential treatment, and malicious incompatibility." The negative consequences of UCIP are significant. On the one hand, it damages governments’ credibility and platform users’ rights while disrupting market competition. On the other hand, large internet platforms have a more potent competitive edge. This advantage has led to the closure of many innovative small platforms in unfair competition scenarios, stifling industry innovation and potentially fostering market monopolies. UCIP has garnered widespread attention from governments, industry enterprises, users, and other groups [5, 6]. There are typical cases of UCIP with far-reaching impacts, as shown in Table 1.

**Table 1:** Typical cases of UCIP.

Internet platforms	Time	Details	Result
Amazon	July 2023	Reached private agreement with Apple to restrict Apple competitors from publishing and selling products on the platform	Spanish antitrust regulator CNMC fined Amazon €50.5 million
Shein	July 2023	Shein uses its market dominance to force clothing manufacturers to sign exclusive agreements to prevent them from working with Temu	Litigation stage, no result
DoorDash	May 2023	Use technical means to identify different terminal users and charge additional fees to iPhone users	Litigation stage, no result
Ctrip	December 2021	Use technical means to identify different user consumption levels and charge additional fees to users with high consumption levels	Compensate consumers 3 times for losses
Alibaba	November 2020	Taking advantage of market dominance to force platform merchants to choose only one between Alibaba or competitor platforms	China Market Supervisory Bureau fined Alibaba 18.228 billion
Google	June 2017	Abuse of search engine dominance to provide illegal advantages to its comparison shopping services	European Commission fined Google €242 million

As core participants in the global digital platform economy, the supervisory systems for UCIP in China and the United States have become a focal point of attention worldwide. Initially, there were significant differences in UCIP supervisory philosophies between China and the United States due to differences in political systems and national conditions. UCIP regulation in the United States follows a down-up philosophy, centers on the internet platform, and focuses on industry self-discipline and social supervision [7]. In contrast, China’s UCIP regulation follows an up-down philosophy, with governments playing a central role, emphasizing administrative legislation and government intervention [8]. UCIP regulation covers various aspects, including politics, economics, and society. With the rapid iterative updates of business models, technical systems, and organizational models of internet platforms, the complexity of supervision has dramatically increased. As a result, the UCIP supervisory systems in China and the United States face some limitations in their practical application. As China’s digital platform economy market capacity increases rapidly, internet platforms have reached tens of millions. The up-down supervisory mode has proven increasingly inadequate, facing challenges such as a lack of supervisory resources, low supervisory effectiveness, and high supervision costs [9]. Therefore, China has actively learned from the strengths of the bottom-up supervisory mode. This includes introducing industry self-regulation and social supervision elements, aiming to build a diverse and coordinated UCIP supervisory system [10]. The multiple collaborative supervision system still centers around governments but also incorporates the participation of industry associations and platform users. On the one hand, governments engage in cooperation for evidence collection and joint law enforcement. They utilize the human and technical resources of industry associations, such as internet associations, E-commerce associations, and internet industry alliances. This serves as a significant supplement to supervisory resources, thereby enhancing supervisory efficiency and reducing supervision costs [11]. On the other hand, the government regards user reports as essential sources of information for investigating UCIP violations, emphasizing the significant role of social supervision [12].

The research contributions of this paper are as follows: (1) Building a UCIP "multi-agent co-governance" system led by governments and participated by industry associations and platform users for the first time. (2) Applying evolutionary game theory to explore the impact mechanism of industry association and platform user participation on the strategic choices of governments and internet platforms. (3) Sorting out the stages of China’s UCIP supervision and matching it with the evolutionary game model results significantly enhances the model’s practical significance.

The research framework of this article is as follows: The second part sorts out UCIP-related research, analyzes research flaws, and proposes research innovations; the third part raises research questions, constructs the evolutionary game models of "unitary supervision" and "multi-agent co-governance," and conducts model solution and analysis; the fourth part conducts model simulation analysis through MATLAB; the fifth part conducts model-to-reality matching analysis. The sixth part presents research conclusions, implications, and summarizes the research shortcomings.

## Literature review

### The UCIP “unitary supervision” mode

Currently, UCIP regulation has attracted many scholars. Some literature has studied the shortcomings of UCIP supervisory policies from the perspectives of illegal identification [13], breadth of application [14], and division of responsibilities [15]. Moreover, a legal system to protect consumer rights and interests has been established at the legislative, law enforcement, and judicial levels. Such studies have a relatively microscopic perspective and fail to answer how governments effectively supervise UCIP. Therefore, more research is shifting toward a macro perspective, examining the government’s UCIP supervisory system and its efficiency. Early studies analyzed supervision experiences from other industries and employed the theoretical concept of "unitary supervision" to construct UCIP government supervisory systems [16]. For instance, Pang S et al. built a static game model between governments and P2P platforms, conducted an empirical analysis based on data from 18 P2P platforms, and explored strategies and paths to enhance government supervision efficiency [17]. Hong Y and Xu J selected Alibaba as a typical analysis object, conducted a systematic study on the Chinese government’s supervision of e-commerce platforms, and proposed regulatory optimization strategies from the government’s perspective [18]. However, the shortcomings of the "unitary supervision" mode have gradually become apparent due to the fast iteration of the business models, technological paradigms, and organizational forms of internet platforms [19]. It makes optimization and improvement of the supervisory system a research focus.

### The UCIP “multi-agent co-governance” mode

In recent years, collaborative governance theory has been continuously improved [20, 21]. Its application in various fields has been deepened, providing new insights for optimizing the UCIP supervisory system. Relevant research has gradually shifted from "unitary supervision" to "multi-agent co-governance." He H and Zhang B believe that unitary government supervision has limitations. These limitations arise due to the large number of internet platforms and extremely complex transaction information, such as insufficient human resources and high financial pressure. Therefore, the government must rely on the auxiliary supervision of third-party institutions, the public, and consumers to supervise internet platforms effectively [22]. You C conducted an in-depth analysis of China’s e-commerce platform supervision regulations and constructed a collaborative supervision system for e-commerce platforms in the upcoming era [23]. Finck M believes that the traditional "unitary supervision" mode is not suitable for internet platform supervision and verifies the superiority of the "multi-agent co-governance" mode through comparative analysis [24]. Liu Y and Zeng W built a collaborative supervision system to address the problems of lack of effectiveness, impact on fairness, and imbalance of power allocation in the supervision of internet financial platforms [25, 26]. Scholars also believe that collaborative governance extends beyond the internal coordination of governments, the social forces should be added to the collaborative governance system. Nie H and Han X used public media and industry associations as supplementary forces for government supervision and built a data monopoly supervision system with collaborative governance. It improves supervision effectiveness in unfair competition, such as price discrimination and user privacy infringement [27]. Liu J and Geng C noted that the traditional decentralized supervision mode has low applicability to the platform economy. Consumers, industry associations, external professional institutions, and other subjects should be asked to participate in supervision efforts [28]. Wei X found that the "unitary supervision" mode has significant difficulties identifying the legitimacy of supervisory objects, dividing supervisory agencies’ functions, and selecting supervision tools. Introducing platform users, industry associations, business representatives, and other entities into internet platform governance can reduce governance costs and improve governance efficiency [29].

### The application of evolutionary game theory in internet platform supervision

Internet platforms may ignore social responsibilities and adopt illegal business practices due to economic interests. As representatives of public power, governments’ supervisory behavior is critical to ensuring internet platform companies comply with government regulations. Therefore, existing research mostly takes governments and internet platforms as game subjects to explore the strategic interaction mechanism. Liu H designed an evolutionary game model between governments and internet financial platforms. The results show that increasing the intensity of punishment will help accelerate the evolution of the game behavior to audit supervision and compliance [30]. Ma W F constructed an evolutionary game model between government departments and online ride-hailing platforms and discovered that achieving Pareto optimal equilibrium is challenging at this stage. However, reducing government supervision costs and increasing punishment intensity will increase the probability of the game system evolving to Pareto optimality [31]. Scholars are gradually introducing third-party entities, such as consumers, into the regulatory system. Shen L et al. found that only when the government’s punishment intensity is higher than the additional supervision costs can e-commerce platforms’ unethical behavior be effectively curbed. At the same time, the study also showed that enhancing consumer participation can improve the government supervision efficiency [32]. According to Li C et al., an internet e-commerce platform will choose a price discrimination strategy when its income exceeds the punishment cost. Consumers can inhibit internet e-commerce platforms’ reputation through social exposure and effectively constrain their price discrimination behaviors [33]. In addition, based on traditional evolutionary game theory, some scholars have also introduced psychological theory to improve internet platform supervision effectiveness. Wu B et al. integrated prospect theory and evolutionary game theory to study the impact mechanism of government supervision and consumer participation on price discrimination behavior on e-commerce platforms [34]. Zhao J et al. built an evolutionary game model that replaced the expected utility function with prospect value [35] to address the internet rumor problem during COVID-19. The above research has laid a solid theoretical foundation for applying evolutionary game theory to UCIP.

The above scholars have studied UCIP supervision from different angles, but limitations still could be improved. (1) Although the auxiliary supervisory role of platform users, industry associations, and other entities is emphasized, the mutual relationships in the supervisory system need to be clearly explained. (2) Most studies carry out analysis from a qualitative and static perspective, and there are still few quantitative and dynamic methods for analysis. (3) A small amount of quantitative research focuses on model construction and solution. It lacks matching analysis between model results and real-life situations, resulting in the model’s insufficient practical significance. In response to the above shortcomings, this paper makes the following improvements and innovations: (1) The UCIP "multi-agent co-governance" system is constructed, and the behavioral relationships among various subjects are clarified. (2) Quantitative and dynamic evolutionary game theory is applied to construct a game model between governments and internet platforms. (3) the Chinese government’s supervision stage of UCIP is explained and matched with the model’s multiple evolutionary stabilization strategies to enhance the model’s practical significance.

This paper’s research objectives are as follows. First, the UCIP "multi-agent co-governance" supervisory system is constructed to explore the participation mechanism of industry associations and platform users. Second, in conjunction with the evolution of UCIP regulations in China, we identify the direction and strategies for future UCIP regulation by the Chinese government.

This paper answers four questions:

1. What are the respective positioning, goals, and interest demands of governments, internet platforms, industry associations, and platform users in the "multi-agent co-governance" system? What kind of game relationship have they created?
2. Compared with the "unitary supervision" mode, what optimizations and improvements have been made in the game model of the "multi-agent co-governance" mode? How has the effectiveness of government supervision been improved?
3. What is the dynamic game decision-making process between governments and internet platforms? How does the participation of industry associations and platform users affect the decision-making of both subjects?
4. What critical parameters affect the strategic choices of governments and internet platforms? In what conditions does the game system evolve towards an ideal state?

## Problem description and model building

### Methodological basis

Evolutionary game theory originated from evolutionary biology. Its core connotation is that bounded rationality determines that game participants cannot reach a stable equilibrium state in a game. This requires a long-term dynamic adjustment process. Evolutionary game theory solves multi-party game problems [36]. Evolutionary game theory assumes that game players in a system possess limited rationality and learning abilities. Players cannot find the optimal strategy through a single round of games due to their limited rationality. Instead, they must continuously learn, imitate, and refine their strategies to discover the most appropriate decision that maximizes their interests. Evolutionary game theory revolves around two core concepts: evolutionarily stable strategies (ESS) and the replicator dynamic equation. ESS is the stable state finally reached by the strategy selected by the players. The definition of the replicator dynamic equation is as follows: $F(k)=\frac{dx_{k}}{dt}=x_{k}[u(k,s)-u(s,s)],k=1,2,3,\ldots .K$.

x_k_ represents the proportion of individuals in a population adopting strategy k. u(k,s) is the fitness of adopting strategy k. u(s,s) is the average fitness in the population. k represents different strategies, and K is the total number of strategies. The definition of ESS is as follows: if s* is an ESS, then s* satisfies the following two basic conditions:

(1) When s* forms a Nash equilibrium, for any s, u(s*,s*)>u(s*s);

(2) If s* ≠ s, satisfies u(s*,s*) = u(s*,s), then u(s*,s)>u(s,s).

The evolutionary game model must meet two conditions: (1) the players are groups rather than individuals. (2) The players’ strategies are subject to dynamic changes, and they continually adjust their strategies and enhance their expected payoffs through multiple rounds of gameplay. In the context of UCIP regulation, the players are governments and internet platforms. Moreover, UCIP supervision is not a fixed state. The game players’ strategy choices vary at different time stages. Therefore, applying the evolutionary game model to UCIP supervision is applicable.

### Game model of the "unitary supervision" mode

#### Problem description

**Fig 1:**
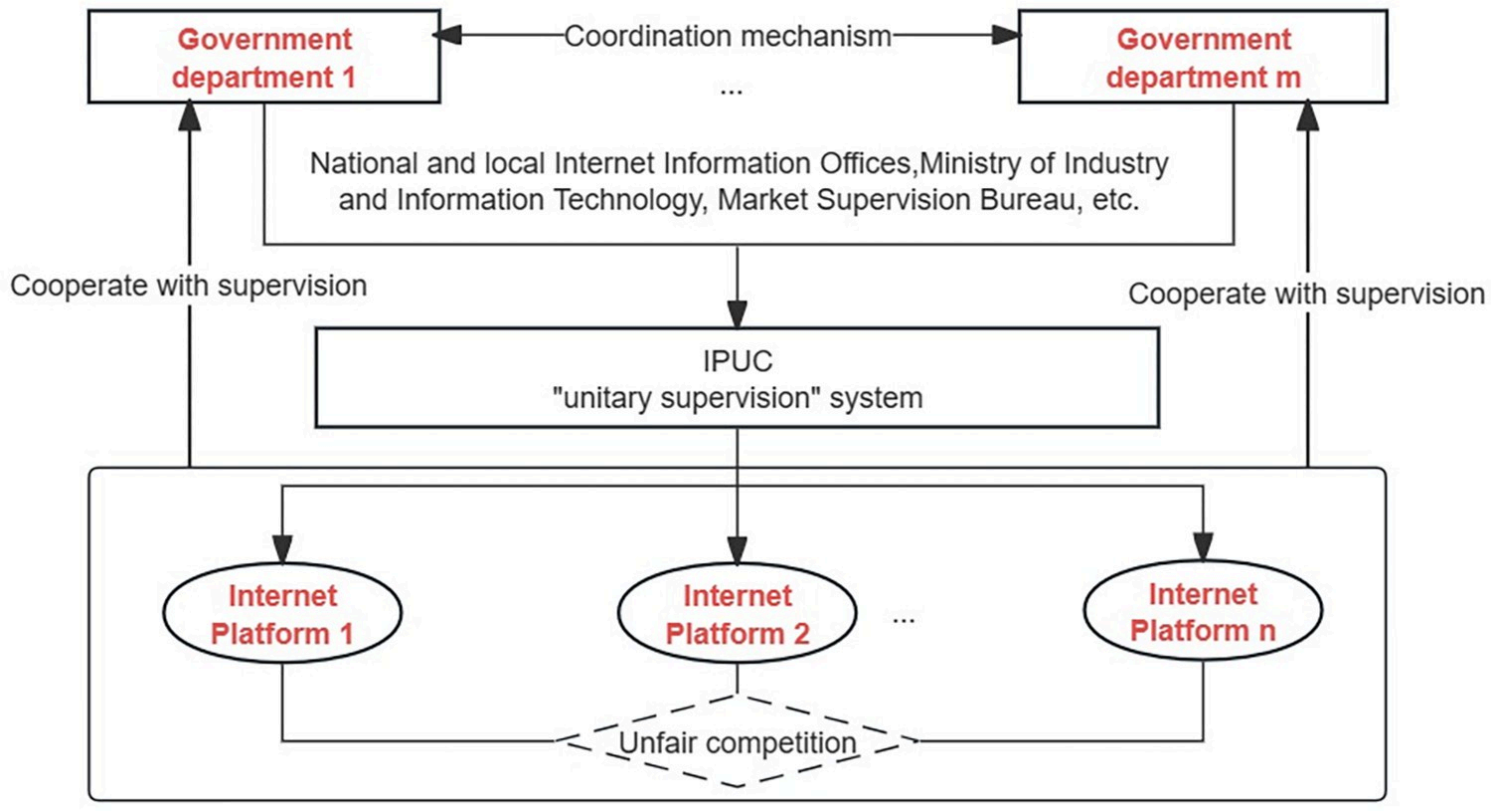
The "unitary supervision" system of UCIP.

This is a basic model. The model considers two game subjects: governments and internet platforms. Internet platforms are profitable organizations in the market, including e-commerce, social networking, short videos, live broadcasts, and takeout platforms. Driven by economic interests, internet platforms with "economic man" characteristics can easily use their technology, scale, and information advantages to conduct unfair competition activities. As representatives of public power, governments include the national and local Ministry of Industry and Information Technology, the Cyberspace Administration, and the Market Supervision Bureau. Governments are supervisory leaders in UCIP and are responsible for formulating and implementing regulation policies and restricting UCIP behavior. The game relationship under the "unitary supervision" mode is shown in Fig 1.

#### Game hypothesis and parameter setting

**Hypothesis 1:** The strategy set of governments is positive supervision and negative supervision. The proportion of positive supervision strategy is x(0≤x≤1), and the proportion of negative supervision strategy is 1−x. The strategy set of internet platforms is fair competition and unfair competition. The proportion of fair competition strategy is y(0≤y≤1), and the proportion of unfair competition strategy is 1−y.

**Hypothesis 2:** The basic operating income of the internet platform is d, and the additional income obtained by the unfair competition strategy is Δd. For example, suppress competitors by " exclusive monopoly " to increase market share, increase product and service prices through "price discrimination" to gain additional profits, and sell user privacy for profit. The cost of unfair competition input is c. The cost of implementing unfair competition is meager because of the advantages of the internet platform in data, technology, and human resources. Because c≪Δd, this paper assumes that c is negligible.

**Hypothesis 3:** The benefits governments obtain from positive supervision are S. It includes political achievements brought about by standardizing the economic order of the platform, improvement of credibility, and rewards from superior departments. The supervision cost is $C=C_{0}+\frac{T^{2}}{2k}(0\leq T\leq 1)$, composed of fixed cost C_0_ and variable cost $\frac{T^{2}}{2k}$. T is the supervision intensity, and k represents the government supervision capability. The larger the parameter k is, the stronger the supervision ability. The smaller the parameter T is, the lower the supervision intensity, and the supervision cost.

**Hypothesis 4:** The information level I_1_(0≤I_1_≤1) determines the information asymmetry between the governments and the internet platforms. The probability u(0≤u≤1) of being investigated and punished for UCIP is determined by parameters I_1_ and T. For convenience of analysis, set u = TI_1_. The two subjects are in a state of information symmetry when T = 1 and I_1_ = 1. The government can 100% investigate and deal with UCIP.

**Hypothesis 5:** The social welfare brought to the government is ω when the internet platforms adopt the fair competition strategy. The positive incentive given by governments to the internet platforms is θω. The parameter θ is the incentive coefficient, such as favorable policies, tax breaks, subsidies, and special funds. The social welfare loss to the government is v when the internet platforms adopt the unfair competition strategy. If the government’s supervision is successful, the internet platform will be punished by z (fines, cease operations). Based on the above hypothesis, the strategic payment matrix for constructing the “unitary supervision” mode is shown in Table 2.

**Table 2:** The strategy interaction payment matrix of the " unitary supervision " mode.

Participants			Internet platform	
			Fair competition y	Unfair competition 1-y
Government	Positive supervision x	$\left[S-{(C}_{0}+\frac{T^{2}}{2k})+\omega -\theta \omega ,d+\theta \omega \right]$	$\left[S-{(C}_{0}+\frac{T^{2}}{2k})-v,d+\Delta d-TI_{1}z\right]$	
	Negative supervision 1-x	(ω,d)	(−v,d+Δd)	

#### Model solving

According to the strategy interaction payment matrix, the expected returns of both subjects under different strategy choices can be calculated. Then, the replication dynamic equation can be obtained.

If the governments choose the "positive supervision" strategy, its expected returns are:

$$E_{x}=y\left[S-{(C}_{0}+\frac{1}{2k}T^{2})+\omega -\theta \omega \right]+(1-y)\left[S-{(C}_{0}+\frac{1}{2k}T^{2})-v\right]$$ (1)

If the governments choose the "negative supervision" strategy, its expected returns are:

$$E_{1-x}=y\omega +(1-y)(-v)$$ (2)

If the internet platforms choose the "fair competition" strategy, its expected returns are:

$$E_{y}=x(d+\theta \omega )+(1-x)d$$ (3)

If the internet platforms choose the "unfair competition" strategy, its expected returns are:

$$E_{1-y}=x[d+\Delta d-TI_{1}z]+(1-x)(d+\Delta d)$$ (4)

According to Formulas (1)–(4), the replication dynamic equations of governments and internet platforms are shown in Formulas (5) and (6).


$$F(x)=\frac{dx}{dt}=x(1-x)\left\{[S-{(C}_{0}+\frac{1}{2k}T^{2})]-\theta \omega y\right\}$$ (5)

$$F(y)=\frac{dy}{dt}=y(1-y)[-\Delta d+(\theta \omega +TI_{1}z)x]$$ (6)

The replication dynamic Eqs (5) and (6) reflect the evolutionary process of the strategy choice of governments and internet platforms. According to the replication dynamic equations, the Jacobian matrix of the game system can be obtained.


$$J=\left[\begin{array}{cc} (1-2x)\left\{[S-{(C}_{0}+\frac{1}{2k}T^{2})]-\theta \omega y\right\} & -x(1-x)(\theta \omega )\\ y(1-y)(\theta \omega +TI_{1}z) & \left(1-2y)[-\Delta d+(\theta \omega +TI_{1}z)x\right] \end{array}\right]$$ (7)

**Proposition 1:** There are four equilibrium points (0,0),(0,1),(1,0),(1,1) in the dynamic game system S_0_. Let $x_{0}=\frac{\Delta d}{\theta \omega +TI_{1}z},y_{0}=\frac{\left[S-{(C}_{0}+\frac{1}{2k}T^{2})\right]}{\theta \omega }$, if 0<x_0_<1,0<y_0_<1,(x_0_,y_0_) is also equilibrium point.

**Proof:** According to the differential equation stability theorem, referring to the solution method of Li S et al. [37] and Chen W et al. [38]. The equilibrium point of the dynamic game system S_0_ should satisfy the replication dynamic Eqs (5) and (6). That is, F(x) = 0, F(y) = 0 and (x,y)∈[0,1]×[0,1]. Therefore, it is clear that (0,0),(0,1),(1,0),(1,1) are the equilibrium points of the game system. By solving Eqs (5) and (6), (x_0_,y_0_) is also equilibrium point when 0<x_0_<1,0<y_0_<1. The proposition 1 is proved.

**Proposition 2:** There are the following situations: the ESS of the system is (0,0) when k<k_0_ and z<z_0_ (or z>z_0_). The ESS of the system is (1,0) when k_0_<k<k_1_ (or k>k_1_) and z<z_0_. The system does not have ESS when k_0_<k<k_1_ and z>z_0_. The ESS of the system is (1,1) when k>k_1_ and z>z_0_.

**Proof:** The equilibrium point obtained by solving the dynamic equation is not necessarily the game system’s ESS. According to the method proposed by Friedman [39], the equilibrium point is ESS when the Jacobian matrix corresponding to the equilibrium point satisfies the conditional Formula (8). By judging the values of each equilibrium point Trj and Det through the Jacobian matrix, the ESS of the game system S_0_ and the satisfied conditions are shown in Table 3.


$$Trj=a_{11}+a_{22}<0,Det=\left|\begin{array}{cc} a_{11} & a_{12}\\ a_{21} & a_{22} \end{array}\right|>0$$ (8)

For the convenience of analysis, let $z_{0}=\frac{\Delta d-\theta \omega }{TI_{1}},k_{0}=\frac{T^{2}}{2(S-C_{0})},k_{1}=\frac{T^{2}}{2(S{-C}_{0}-\theta \omega )}$. The proposition 2 is proved.

**Table 3:** The ESS and parameter value conditions of the "unitary supervision" mode.

equilibrium point	TrJ	DetJ	Condition 1	Condition 2
A(0,0)	−	+	$\left[S-{(C}_{0}+\frac{1}{2k}T^{2})\right]<0$	Δd−θω>TI_1_z or Δd−θω<TI_1_z
B(1,0)	−	+	$0<\left[S-{(C}_{0}+\frac{1}{2k}T^{2})\right]<\theta \omega \;or\left[S-{(C}_{0}+\frac{1}{2k}T^{2})\right]>\theta \omega$	Δd−θω<TI_1_z
C(x_0_,y_0_)	uncertain	−	$0<\left[S-{(C}_{0}+\frac{1}{2k}T^{2})\right]<\theta \omega$	Δd−θω<TI_1_z
D(1,1)	−	+	$\left[S-{(C}_{0}+\frac{1}{2k}T^{2})\right]>\theta \omega$	Δd−θω<TI_1_z

### Game model of "multi-agent co-governance" mode

#### Problem description

**Fig 2:**
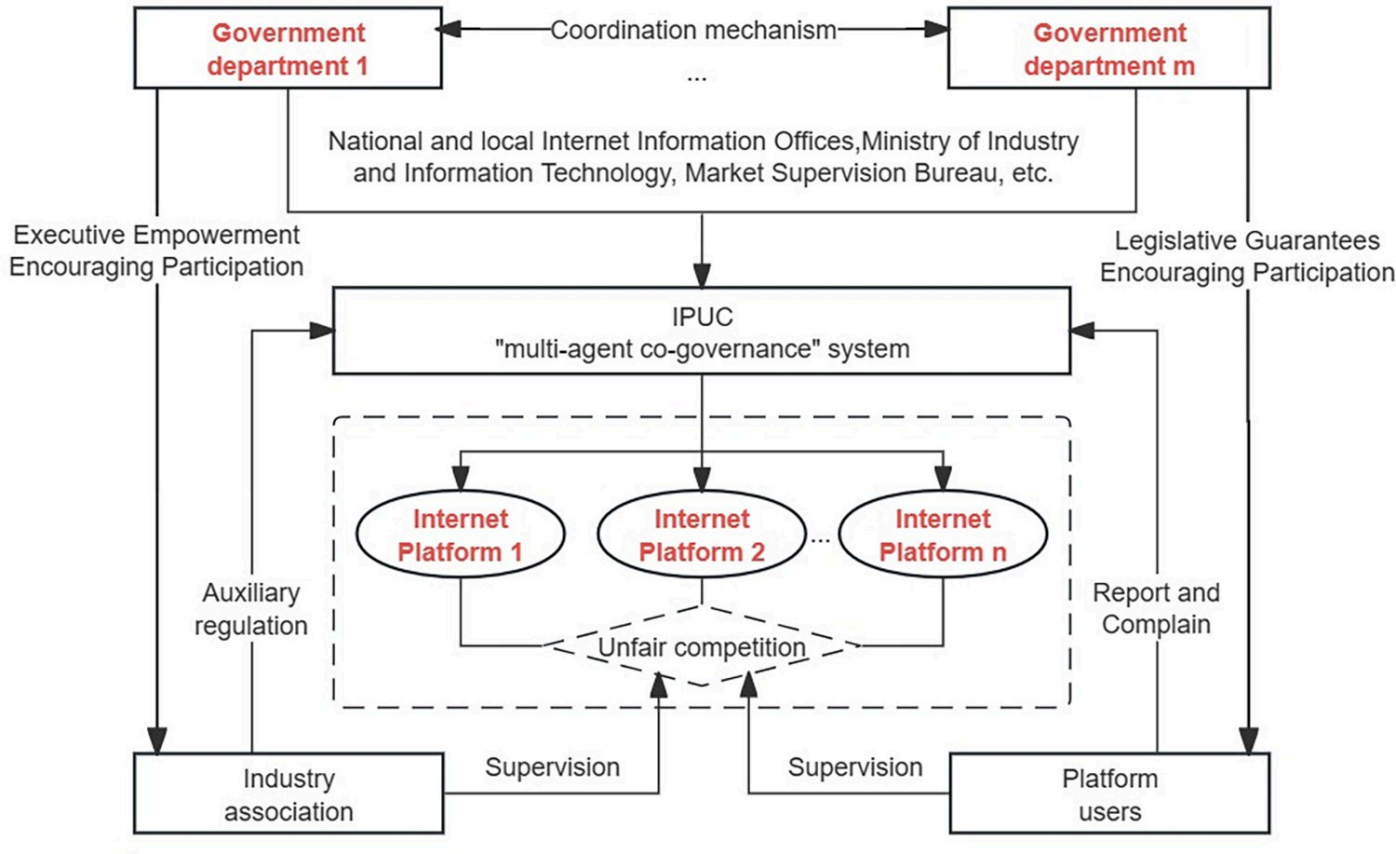
The “multi-agent co-governance " system of UCIP. Compared with the "unitary supervision" mode, the "multi-agent co-governance" mode incorporates industry associations and platform users into the supervision system. The "multi-agent co-governance" mode emphasizes the diversification of supervisory entities and the coordination of the supervisory process. A synergistic effect is generated through the functional complementation and benign interaction of various entities in the system, thereby improving supervisory efficiency.

This is an improved model. Compared with unfair competition in traditional industries, new types of unfair competition derived from the internet have high dynamics, complexity, and concealment characteristics. Thus, it reduces the applicability of the traditional “unitary supervision” mode. Furthermore, due to governments’ limited supervision resources and capabilities, the "unitary supervision" mode often suffers from "supervision failure" problems. Based on the theoretical framework of collaborative governance, this paper constructs a systematic and flat "multi-agent co-governance" system of UCIP, as shown in Fig 2.

Industry associations are third-party social organizations, including national and local internet associations, internet industry alliances, e-commerce industry associations, and webcast industry associations. Platform users, including consumers and merchants, receive internet platforms’ goods and services. On the one hand, industry associations play an auxiliary role and do not directly regulate UCIP. On the other hand, facing powerful governments and internet platforms with capital, technology, and information advantages, platform users are in a relatively weak position in the UCIP supervisory game. Thus, this paper introduces industry associations and platform users into the game system as critical parameters instead of treating them as game subjects.

#### Game hypothesis and parameter setting

As an emerging economic form, the platform economy is more complicated to supervise than traditional industries. Due to the diversification and concealment of UCIP means and methods, governments’ investigation and evidence collection period is long and arduous. This increases supervision costs. Limited supervision resources face significant challenges. By building a "multi-agent co-governance" system, industry associations and platform users can participate in UCIP supervision. The participation logic is as follows.

1. Industry associations: First, industry association participation can share part of government supervision costs and effectively alleviate the shortage of administrative supervision resources. For example, assisting the government with investigation, evidence collection, and analysis. The governments can also entrust highly professional work (industry norms, industry standards, special inspections, and rectifications) to industry associations for implementation. Second, the industry associations have information, knowledge, and technology advantages. Thus, the industry associations can more easily monitor information about UCIP and fill in governments’ information blind spots through information sharing. This reduces information asymmetry between governments and internet platforms to achieve precise positioning and supervision goals.
2. Platform users: First, platform users, as direct victims of UCIP, can report and complain to governments through various channels, improving government information levels. Second, the government’s disclosure of unfair competition behavior through official websites and news media will diminish the company’s social reputation. Consequently, it may decrease platform users. At the same time, the platform market has network externalities. The decreasing platform users will inevitably reduce the matching efficiency and service quality of the platform, thereby reducing platform revenue. Therefore, under the combined effect of reputation mechanisms and network externalities, platform users can use "leave the platform" to deter internet platforms’ unfair competition behavior.

Therefore, based on the assumptions of the "unitary supervision" mode, the new assumptions are as follows:

**Hypothesis 6:** As the participation level of industry associations increases and replaces part of the government’s supervision functions, the supervision cost will gradually decrease. The supervision cost function could be rewritten as $C=C_{0}+\frac{T^{2}(1-ep)}{2k}$. Where p is the participation degree of industry associations, e is the power space. The increase in p and e will reduce variable supervision costs. Since industry associations are nongovernmental organizations, governments will limit the scope of power of industry associations to prevent any potential abuse of power. At the same time, industry associations obtain unfair competition information on internet platforms through their advantages in knowledge and technology and share the information I_2_ with governments. If the government chooses the negative supervision strategy, it will lose credibility when industry associations participate. The credibility loss is assumed to be pm, which increases with industry participation.

**Hypothesis 7:** Platform users share information I_3_ with governments through reporting and complaints after discovering UCIP. However, as vulnerable groups, platform users cannot fully share their information with governments. The parameter ε represents the information sharing level affected by sharing channels and costs. After the government successfully investigates and deals with UCIP, it announces to the public through official websites, media, and other channels. The negative utility brought to the internet platform by platform users "leave the platform" is qαΔτ. The parameter α is the conversion coefficient between the number of users and platform revenue. The parameter Δτ is the number of users on the platform. The parameter q is the degree of user participation. The larger the q, the lower the user stickiness of the platform, the more sensitive to UCIP, and the greater the probability of "leave the platform."

**Hypothesis 8:** After industry associations and platform users share information, the information level mastered by governments increases to I. I = f(I_1_,I_2_,εI_3_), satisfying $I_{i}\leq I\leq 1,(i=1,2,3),\frac{\partial I}{\partial I_{1}}>0,\frac{\partial I}{I_{2}}>0,\frac{\partial I}{I_{3}}>0,\frac{\partial I}{\varepsilon }>0$. Based on the above hypotheses, the strategic payment matrix for constructing the “multi-agent co-governance” mode is shown in Table 4.

**Table 4:** The strategy interaction payment matrix of the "multi-agent co-governance" mode.

Participants			Internet platform	
			Fair competition y	Unfair competition 1-y
Government	Positive supervision x	$\left[S-{(C}_{0}+\frac{T^{2}(1-ep)}{2k})+\omega -\theta \omega ,d+\theta \omega \right]$	$\left[S-{(C}_{0}+\frac{T^{2}(1-ep)}{2k})-v,d+\Delta d-TI(z+q\alpha \Delta \tau )\right]$	
	Negative supervision 1-x	(ω,d)	(−v−pm,d+Δd)	

#### Model solving

According to the strategy interaction payment matrix, the expected returns of both subjects under different strategy choices can be calculated. Then, the replication dynamic equation can be obtained.

If the governments choose the "positive supervision" strategy, its expected returns are:

$$E_{x}=y\left[S-{(C}_{0}+\frac{T^{2}(1-ep)}{2k})+\omega -\theta \omega \right]+(1-y)\left[S-{(C}_{0}+\frac{T^{2}(1-ep)}{2k})-v\right]$$ (9)

If the governments choose the "negative supervision" strategy, its expected returns are:

$$E_{1-x}=y\omega +(1-y)(-v-pm)$$ (10)

If the internet platforms choose the "fair competition" strategy, its expected returns are:

$$E_{y}=x(d+\theta \omega )+(1-x)d$$ (11)

If the internet platforms choose the "unfair competition" strategy, its expected returns are:

$$E_{1-y}=x[d+\Delta d-TI(z+q\alpha \Delta \tau )]+(1-x)(d+\Delta d)$$ (12)

Thus, according to Formulas (9)–(12), the replication dynamic equations of governments and internet platforms are shown in (13),(14).


$$F(x)=\frac{dx}{dt}=x(1-x)\left\{[S-{(C}_{0}+\frac{T^{2}(1-ep)}{2k})+pm]-y[\theta \omega +pm]\right\}$$ (13)

$$F(y)=\frac{dy}{dt}=y(1-y)\{-\Delta d+[\theta \omega +TI(z+q\alpha \Delta \tau )]x\}$$ (14)

Referring to the game model solving and proof process of the "unitary supervision" mode, we can obtain the following Proposition.

**Proposition 3:** There are four equilibrium points (0,0),(0,1),(1,0),(1,1) in the dynamic game system S_1_. Let $x_{1}=\frac{\Delta d}{\theta \omega +TI(z+q\alpha \Delta \tau )},y_{1}=\frac{\left[S-{(C}_{0}+\frac{T^{2}(1-ep)}{2k})+pm\right]}{\theta \omega +pm}$, if 0<x_1_<1,0<y_1_,<1,(x_1_,y_1_) is also equilibrium point. Through solving the Jacobian matrix, the ESS of the game model under "multi-agent co-governance" mode and the conditions satisfied are shown in Table 5.

**Table 5:** The ESS and parameter value conditions of the " multi-agent co-governance " mode.

Equilibrium point	TrJ	DetJ	Condition 1	Condition 2
A′(0,0)	−	+	$S-{(C}_{0}+\frac{T^{2}(1-ep)}{2k})+pm<0$	Δd−θω>TI(z+qαΔτ) or Δd−θω<TI(z+qαΔτ)
B′(1,0)	−	+	$0<S-{(C}_{0}+\frac{T^{2}(1-ep)}{2k})+pm<\theta \omega +pm$ or $S-{(C}_{0}+\frac{T^{2}(1-ep)}{2k})+pm>\theta \omega +pm$	Δd−θω>TI(z+qαΔτ)
C′(x_1_,y_1_)	uncertain	−	$0<S-{(C}_{0}+\frac{T^{2}(1-ep)}{2k})+pm<\theta \omega +pm$	Δd−θω<TI(z+qαΔτ)
D′(1,1)	−	+	$S-{(C}_{0}+\frac{T^{2}(1-ep)}{2k})-\theta \omega >0$	Δd−θω<TI(z+qαΔτ)

**Proposition 4:** The game system presents different ESS in the context of the "multi-agent co-governance" mode, with parameters p, q, and z change.

**Proof:** Let $p_{0}=\frac{{2k(C}_{0}-s)+T^{2}}{2km+{eT}^{2}};\;p_{1}=\frac{T^{2}-2k(S-C_{0}-\theta \omega )}{eT^{2}};\;q_{0}=\frac{\Delta d-\theta \omega -TIz}{\alpha \Delta \tau TI};\;z_{0}=\frac{\Delta d-\theta \omega }{TI}-q\alpha \Delta \tau$; modify the parameters in Table 5, as shown in Table 6.

**Table 6:** The ESS and parameter value conditions of the "multi-agent co-governance" mode.

Equilibrium point	TrJ	DetJ	Condition 1	Condition 2	
A′(0,0)	−	+	p<p_0_	q>q_0_ or q<q_0_	z>z_0_ or z<z_0_
B′(1,0)	−	+	p_0_<p<p_1_ or p>p_1_	q<q_0_	z<z_0_
C′(x_1_,y_1_)	uncertain	−	p_0_<p<p_1_	q>q_0_	z>z_0_
D′(1,1)	−	+	p>p_1_	q>q_0_	z>z_0_

**Fig 3:**
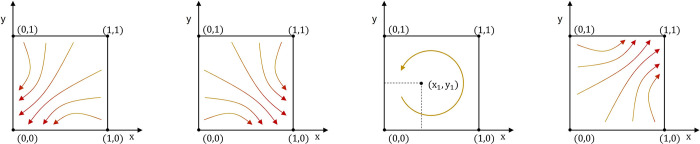
The evolutionary phase diagrams of equilibrium points.

The evolutionary phase diagrams corresponding to each equilibrium point are shown in Fig 3.

### Game model analysis

#### Evolutionary stage analysis

**Proposition 5:** The equilibrium point (0,0) is ESS when p<p_0_ and q∈R (or z∈R). The state is not conducive to the healthy and orderly development of the platform economy. It is an unfavorable period when governments and internet platforms do not work together.

Regardless of platform user participation level (or punishment intensity), when industry association participation level is low, the governments choose the negative supervision strategy. In contrast, the internet platforms choose unfair competition. In this scenario, the supervision benefits are relatively small. Industry associations have limited influence and bear a small share of supervision costs. The government will adopt the negative supervision strategy due to limited supervision capabilities and resources. The internet platforms will frantically pursue economic interests through unfair competition driven by economic interests and due to the lack of external government supervision. As evolutionary time increases, the system eventually evolves toward the equilibrium point (0,0).

**Proposition 6:** The equilibrium point (1,0) is ESS when p>p_0_ and q<q_0_ (or z<z_0_). This state has improved compared with (0,0). The strategic choice of governments has changed to positive supervision. However, due to platform users’ insufficient participation and imperfect government punishment mechanisms, effective UCIP regulation remains unattainable.

The governments adopt the positive supervision strategy, and the internet platforms adopt the unfair competition strategy when the industry association participation is higher than a certain level and platform user participation is low (or the punishment intensity is low). In this scenario, the UCIP is intensifying, and the negative impact on the economy and society is increasing, attracting widespread attention. The governments enhance supervision capabilities by improving supervision technology and methods. At the same time, industry association participation has increased, and supervision costs have dropped significantly. The government will choose a positive supervision strategy.

Due to the low participation of platform users (or the light punishment intensity), it has not significantly impacted the expected returns of internet platforms adopting unfair competition strategies. The internet platforms will still choose the unfair competition strategy. As evolutionary time increases, the system eventually evolves toward the equilibrium point (1,0).

**Proposition 7:** There is no ESS for the system when p_0_<p<p_1_ and q>q_0_ (or z>z_0_). In this state, the strategic choices of the two subjects depend on each other, showing periodic fluctuation characteristics. This is the transition period of the system’s evolution toward the ideal state.

The system has no ESS when industry associations’ participation level is within a specific range and platform users’ participation is high (or the punishment intensity is heavy). In this scenario, various industries are actively carrying out digital transformation, and new business forms and modes have fully emerged. Internet platforms have also increased rapidly and peaked, causing tremendous pressure on government supervision resources. Although the participation level of industry associations has increased and can share part of the supervision costs, it is not enough to support the cost input of "Normalization" supervision. The government will adopt a "campaign-style" supervision strategy. When UCIP risk increases, governments will strengthen supervision. Because of the limited supervision resources, it will have to reduce the supervision intensity, resulting in frequent unfair competition incidents. The governments will continue strengthening the supervision intensity, so back and forth. Due to the increased participation of platform users (or punishment intensity), the expected returns of internet platforms adopting unfair competition strategies will be significantly reduced. The strategic choices of the internet platforms will change with the government’s strategic choices. The proportion of the internet platform group choosing the fair competition strategy is consistent with that of the government group choosing the positive supervision strategy. As evolutionary time increases, the strategic choices of governments and internet platforms become interdependent. When the strategic choices of the other subject change, they will increase their expected returns by changing their strategies. There is no evolutionarily stable strategy.

**Proposition 8:** The equilibrium point (1,1) is ESS when p>p_1_ and q>q_0_ (or z>z_0_). In this state, due to the joint participation of multiple subjects in coordinated supervision, UCIP has been effectively regulated. This is an ideal period.

The governments adopt the positive supervision strategy when industry association and platform user participation are very high (or the punishment intensity is heavy). At the same time, the internet platforms adopt the fair competition strategy. In this scenario, the high participation of industry associations helps governments share many supervision costs, and governments improve their supervision capabilities by further optimizing technologies and methods. Because of the high participation of platform users (or the heavy punishment intensity), the internet platform’s unfair competition returns will be significantly reduced. As evolutionary time increases, the proportion of governments adopting the positive supervision strategy and internet platforms adopting the fair competition strategy increases. The game system eventually evolves toward the equilibrium point (1.1).

**Fig 4:**
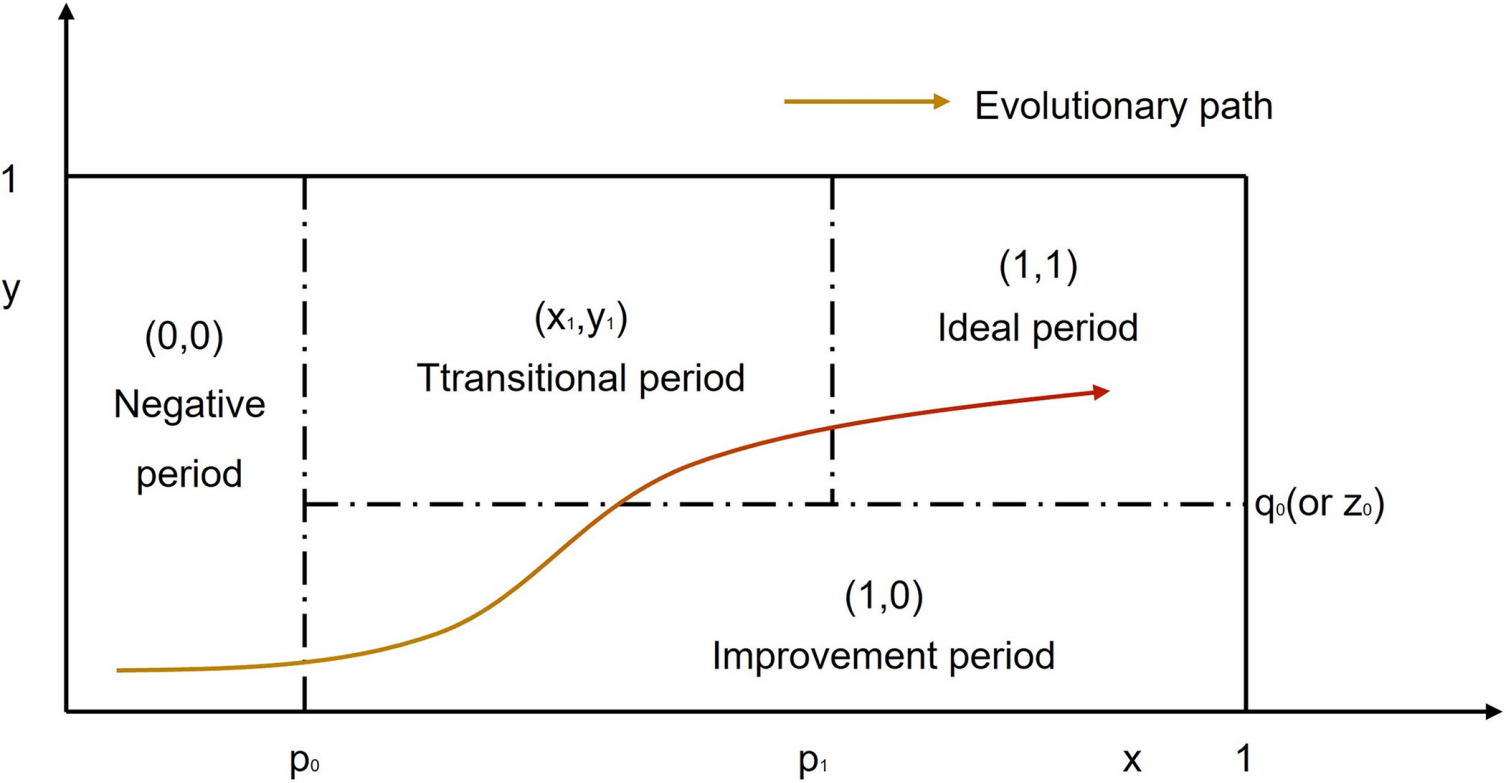
The evolutionary path of UCIP.

Combined with propositions 5–8, the evolutionary path of the game system can be described as (0,0)→(1,0)→(x_1_,y_1_)→(1,1). The evolutionary path is expressed on the same axis as shown in Fig 4.

#### Parameter sensitivity analysis

**Fig 5:**
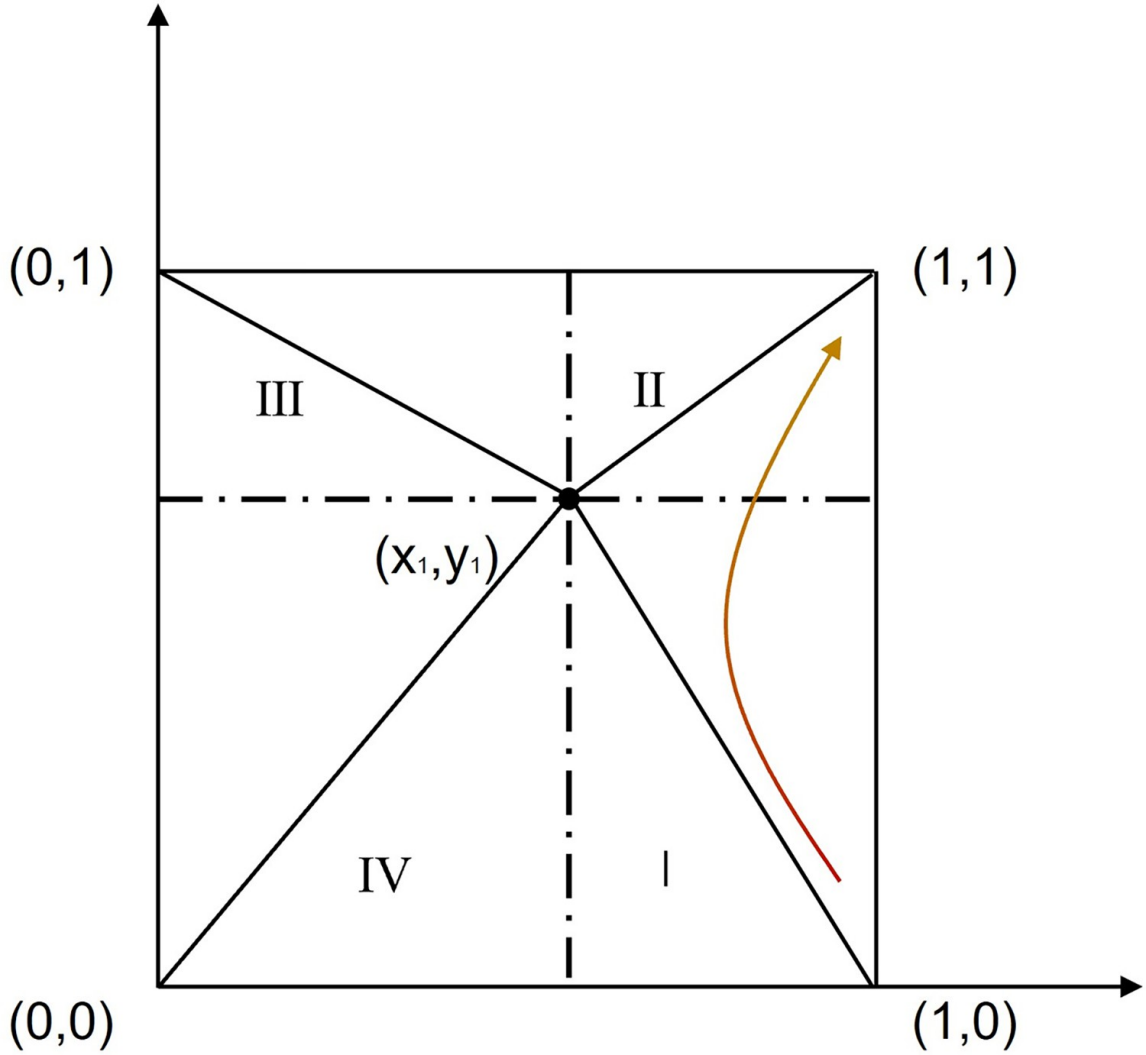
The two-dimensional plan analysis.

When the parameters satisfy p_0_<p<p_1_ and q>q_0_ (or z>z_0_), the game process between governments and internet platforms is challenging to control. It can improve the probability of system evolution to (1,1) by adjusting the value of critical parameters. Place governments and internet platforms on the two-dimensional plane coordinate axis S = {(x,y); 0≤x,y≤1}. The plane S is divided into regions I, II, III, and IV by two dotted lines. When the initial point of the game falls into region I, the system converges to the equilibrium point (1,1). Increasing the area S of region I can guide the system to evolve toward the positive state (1,1). The two-dimensional plane map is shown in Fig 5.

The expression of the area S is:

$$S=[1-\frac{\Delta d}{\theta \omega +TI(z+q\alpha \Delta \tau )}]\frac{\left[S-{(C}_{0}+\frac{T^{2}(1-ep)}{2k})+pm\right]}{\theta \omega +pm}$$ (15)

**Proposition 9:** The higher the degree of industry association participation p, the larger the power space e, which will promote the evolution of the game system to the ideal state (1,1).

**Proof:** The first-order partial derivatives of parameters p and e for S are $\frac{\partial S}{\partial p}>0,\frac{\partial S}{\partial e}>0$, indicating that S is an increasing function of the parameters p and e. That is, an increase in the value of p and e will increase the area of S.

1. Industry association participation p. As a bridge between governments and the market, industry associations are an indispensable subject of the "multi-agent co-governance" system. For example, the United Kingdom and the United States have established P2PFA and MLA industry associations to supervise internet lending platforms and promote self-discipline. Industry associations’ participation in "multi-agent co-governance" still faces problems such as weak resources, imperfect mechanisms, and single modes. It is necessary to remove obstacles to industry associations’ participation and increase their participation level through mechanism reforms and mode innovations. On the one hand, industry associations need to assist governments in formulating supervision policies, supervising internet platforms, and reducing government supervision costs. On the other hand, it is necessary to urge self-discipline in internet platform market entities’ behavior by formulating industry conventions, association rules, and public interest litigation.
2. Power space e. The prerequisite for industry associations to participate in governance is to have a specific power scope before they can play their role in auxiliary supervision. Governments must determine the reasonable "degree" of power space given to industry associations. If the power space is too large, problems such as corruption, interest exchange, and abuse of power will easily arise. However, if the power space is too small, it is not conducive to industry associations’ synergy in the co-governance system. Therefore, the government needs to target industry associations’ role as "synergists." Specific issues that are noncore, highly professional, and in line with industry associations’ capabilities can be properly decentralized and handed over to industry associations for supervision.
  **Proposition 10:** The greater the public preference q, the greater the information sharing level ε, which will promote the evolution of the game system to the ideal state (1,1).
  **Proof:** The first-order partial derivatives of parameters q and ε for S are $\frac{\partial S}{\partial q}>0,\frac{\partial S}{\partial \varepsilon }>0$, indicating that S is an increasing function of parameters q and ε. That is, an increase in the value of q and ε will increase the S area.
3. Platform user participation q. Since platform users do not have much power in the "multi-agent co-governance" system, they cannot directly punish UCIP through coercive means when their rights and interests are damaged. However, platform users have the right to choose the platform and can indirectly restrain the platform by "leave the platform" as a deterrent. Platform user participation depends on many factors, including user stickiness, market share of internet platforms, and damage to interests. Platform user participation differs significantly in different scenarios.
4. Information sharing level ε. After platform users discover UCIP, they can share illegal information with governments through complaints and reports. China has established relatively smooth complaint and reporting platforms, including telephone, email, and online network platforms (the Internet Information Service Complaint Platform, 12315 Platform, 12377 Platform), providing users diversified participation channels.

**Proposition 11:** The greater the punishment z, which will promote the evolution of the game system to the ideal state (1,1). The influence of T on the evolution state is uncertain. There is an optimal value that makes the area of S largest.

**Proof:** The first-order partial derivative of parameters z for S is ∂S/∂z>0. The positive or negative of ∂S/∂T cannot be determined. The area S is a convex function about the parameter T. There is a T_0_ value in the interval [0,1]. When T<T_0_, ∂S/∂T>0, and when T>T_0_,∂S/∂T<0.

1. Supervision intensity T. Under the traditional "unitary supervision" mode, increasing supervision intensity is the primary means for governments to reduce UCIP risk. However, the resulting increase in supervision costs also reduces enthusiasm for supervision. Suppose the government’s supervision intensity is too low. In that case, it will increase the information asymmetry with the internet platform. This, in turn, will be unable to effectively deter its unfair competition behavior, resulting in a less-than-expected supervision effect. Therefore, governments must balance supervision intensity and costs, maintaining supervision at an appropriate level while optimizing means and methods to reduce costs and enhance supervisory effects. At the same time, it is necessary to appropriately disperse supervision powers and enrich complaint reporting channels. This approach ensures the optimal balance between supervision effects and costs by the auxiliary supervision role of industry associations and platform users.
2. Punishment intensity z. Because of the limited rationality of the internet platform, strategy choice is based on maximizing its interests. Suppose the punishment for UCIP is minor until the expected return of the unfair competition strategy is greater than that of the fair competition strategy. In that case, the punishment and restraint mechanism will inevitably fail. The punishment and restraint mechanism can be improved from two aspects. On the one hand, the economic punishment mechanism for internet platform violations should be specified in policies and regulations. On the other hand, notify and publicly criticize the internet platform’s unfair competition behavior. Announce it promptly to the public through government websites and news media to reduce the internet platform’s social reputation.

## Model simulation analysis

TCOM, China’s largest internet travel platform, was listed on Stanak in December 2003. In recent years, TCOM has been suspected of multiple unfair competition behaviors, such as "price discrimination and an exclusive monopoly." (1) In July 2020, a platform user paid 3,000 yuan to book a hotel at TCOM but found the actual price was only 1,400 yuan in the next day. The paid price was more than twice the actual price. Subsequently, the user took TCOM to court, and the court ordered TCOM to compensate the user three times the loss. (2) In October 2021, the weekend hotel reported TCOM’s "exclusive monopoly" and forced platform merchants to choose only one between TCOM and its competitor platforms. The above incidents were widely spread on the Chinese internet, negatively impacting TCOM’s reputation and leading to platform users’ loss. In response to TCOM’s unfair competition, governments (Market Supervision Bureau, Industry and Commerce Bureau) and industry associations (Internet Association, Consumer Association, Payment Association) conducted supervisory investigations on TCOM. In response to TCOM’s unfair competition, governments fined TCOM more than one million yuan.

Because the game model has many parameters, empirical data collection is challenging. This article assigns values to parameters based on the following two principles: (1) Refer to the general assignment methods in the literature in the field of evolutionary games [40–42]; (2) The parameter values satisfy the logical relationships in Tables 3 and 5. The parameter value initialization is shown in Table 7.

**Table 7:** The parameter initialization value.

Parameter	*S*	C_0_	T	ω	Δd	I_1_	m	α	Δτ
Value	5	3	0.1	10	5	0.1	2	0.1	1000

### Evolutionary stage analysis

In this section, MATLAB is used to describe the evolutionary trajectory of the game subject’s behavior in different supervision stages. At the same time, we verify the influence of critical parameter value changes on the game system ESS. The ode45 command is used to solve replication dynamic equations and analyze the changing trend of the positive supervision ratio x and the fair competition ratio y. Set seven initial points: (0.2 0.4), (0.5 0.5), (0.8 0.6), (0.9 0.9), (0.3 0.9), (0.9 0.3), (0.6 0.8).

**Fig 6:**
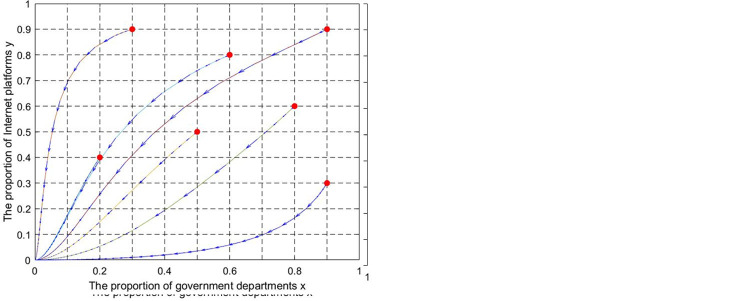
Simulation diagram of the dynamic evolution of the game system (0,0) stable point. (a) The “unitary supervision” mode. (b) The “multi-agent co-governance” mode.

**Fig 7:**
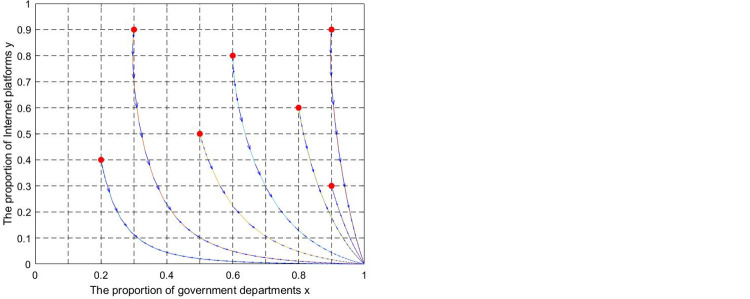
Simulation diagram of dynamic evolution of game system (1,0) stable point. (a) The “unitary supervision” mode. (b) The “multi-agent co-governance” mode.

**Fig 8:**
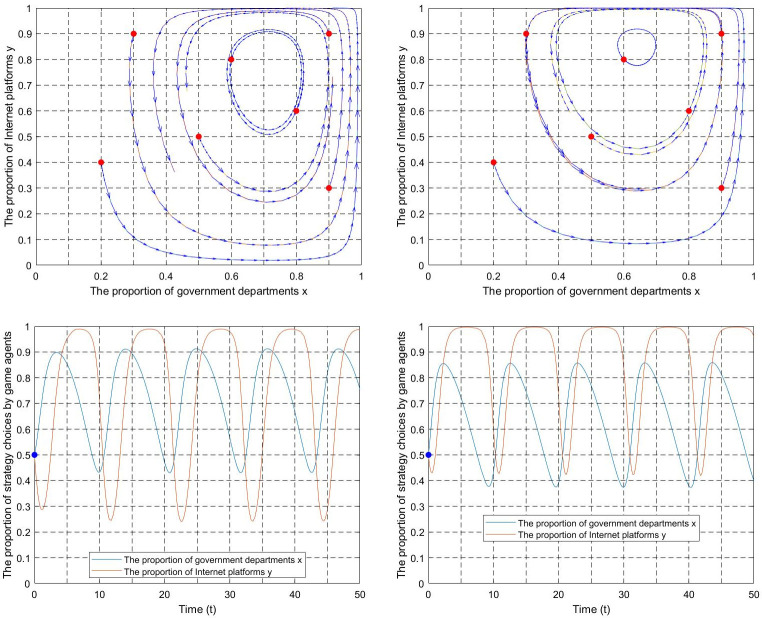
Simulation diagram of dynamic evolution of game system without evolutionarily stable strategy. (a) The “unitary supervision” mode. (b) The “multi-agent co-governance” mode. (c) The “unitary supervision” mode. (d) The “multi-agent co-governance” mode.

**Fig 9:**
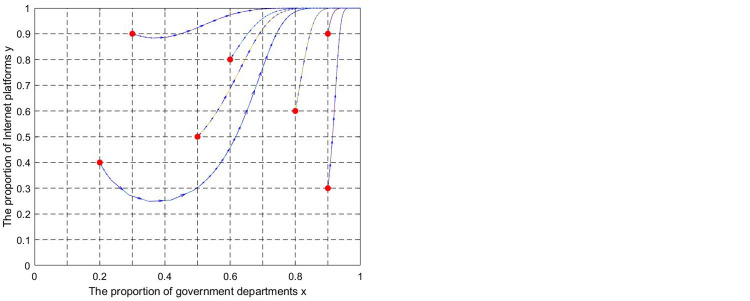
Simulation diagram of dynamic evolution of game system (1,1) stable point. (a) The “unitary supervision” mode. (b) The “multi-agent co-governance” mode.

(1) ESS (0,0). Let k = 0.001, θ = 0.2,z = 5, e = 0.5,p = 0.1, I = 0.2, q = 0.1. The evolutionary trajectory of state A and state A′ is shown in Fig 6. The lines in the simulation graphs represent the evolutionary paths corresponding to different initial points. Regardless of the initial point location, the values of x and y decrease over time. The system eventually evolves toward the (0,0) stable point, which verifies proposition 5. The simulation results show that if government supervision is absent, the internet platform will obtain illegal economic benefits through unfair competition driven by economic interests. Relying on market self-discipline cannot achieve the purpose of UCIP regulation. In this state, industry associations and platform users play a limited role. It cannot change the evolutionary stability strategy of the system, but it slows down the game system’s evolution toward unfavorable results.

(2) ESS (1,0). Let k = 0.01,θ = 0.2,z = 5, e = 0.5,p = 0.2,I = 0.2,q = 0.2, the evolutionary trajectory of state B and state B’ is shown in Fig 7. The lines in the simulation graphs represent the evolutionary paths corresponding to different initial points. Regardless of the initial point location, the value of x increases over time while the value of y decreases. The system eventually evolves toward the (1,0) stable point, which verifies proposition 6. The simulation results show that as the participation of industry associations increases, the supervision cost is effectively reduced, leading governments to choose the positive supervision strategy. However, the internet platforms still adopt the unfair competition strategy due to the lack of effective punitive mechanisms. In this state, increasing the participation of industry associations can only change the strategic choices of governments but cannot change the strategic choices of internet platforms. The game system still evolves toward unfavorable results.

(3) No ESS. Let k = 0.01,θ = 0.2,z = 500, e = 0.5,p = 0.4,I = 0.2,q = 0.4, the evolutionary trajectory of state C and state C’ is shown in Fig 8. The lines in the simulation graphs represent the evolutionary paths corresponding to different initial points. Regardless of the initial point location, the strategic choices of governments and internet platforms exhibit cyclical oscillations, which verifies proposition 7. In this state, the periodic oscillation situation cannot be terminated due to the insufficient participation of industry associations. However, the oscillation amplitude is reduced, the system stability is improved, and the probability of the game system evolving toward (1,1) is increased.

(4) ESS (1,1). Let k = 0.02,θ = 0.1,z = 500, e = 0.8,p = 0.8,I = 0.8,q = 0.6, the evolutionary trajectory of state D and state D′ is shown in Fig 9. The lines in the simulation graphs represent the evolutionary paths corresponding to different initial points. Regardless of the initial point location, the values of x and y increase over time. The system eventually evolves toward the (1,1) stable point, which verifies proposition 8. The simulation results show that maintaining industry association and platform user participation (or punishment intensity) at a relatively high level will lock the game system into an ideal state. In this state, the industry association and platform user participation facilitate the game system’s evolution toward the ideal state and expedite the evolution rate.

According to Figs 6–9, when x and y evolve toward 0, compared with the "unitary supervision " mode, the "multi-agent co-governance" mode corresponds to a longer evolution trajectory and evolution time. However, the result is the opposite when x and y evolve toward 1. This shows that the participation of industry associations and platform users will hinder the evolution of the game system to the equilibrium point (0,0) but promote the evolution of the game system to the equilibrium point (1,1,). Under the "multi-agent co-governance" mode, there is still an evolutionary equilibrium point (0,0). This shows that even if the government adopts a "multi-agent co-governance" mode if the regulatory capacity is weak or the participation of industry associations and platform users is low, the problem of "regulatory failure" may still occur.

It is worth noting that to more intuitively compare the differences in the supervisory effects of the two supervision modes. It is limited that the two modes have a consistent ESS when simulating the values of the parameters in Figs 6–9. There are cases where industry associations and platform users are introduced to achieve a state transition when the "unitary supervision" mode is in a specific state. For such cases, the impact of each critical parameter on the ESS of the game system will be analyzed below.

### Parameter sensitivity analysis

Referring to the parameter assignment principles and methods in the previous section, the initial values of the parameters in this section are shown in Table 8.

**Table 8:** Parameter initialization value table.

Parameter	*S*	C_0_	e	θ	ω	Δd	I	α	Δτ
value	5	1	0.5	0.1	10	5	0.2	0.2	1000

**Fig 10:**
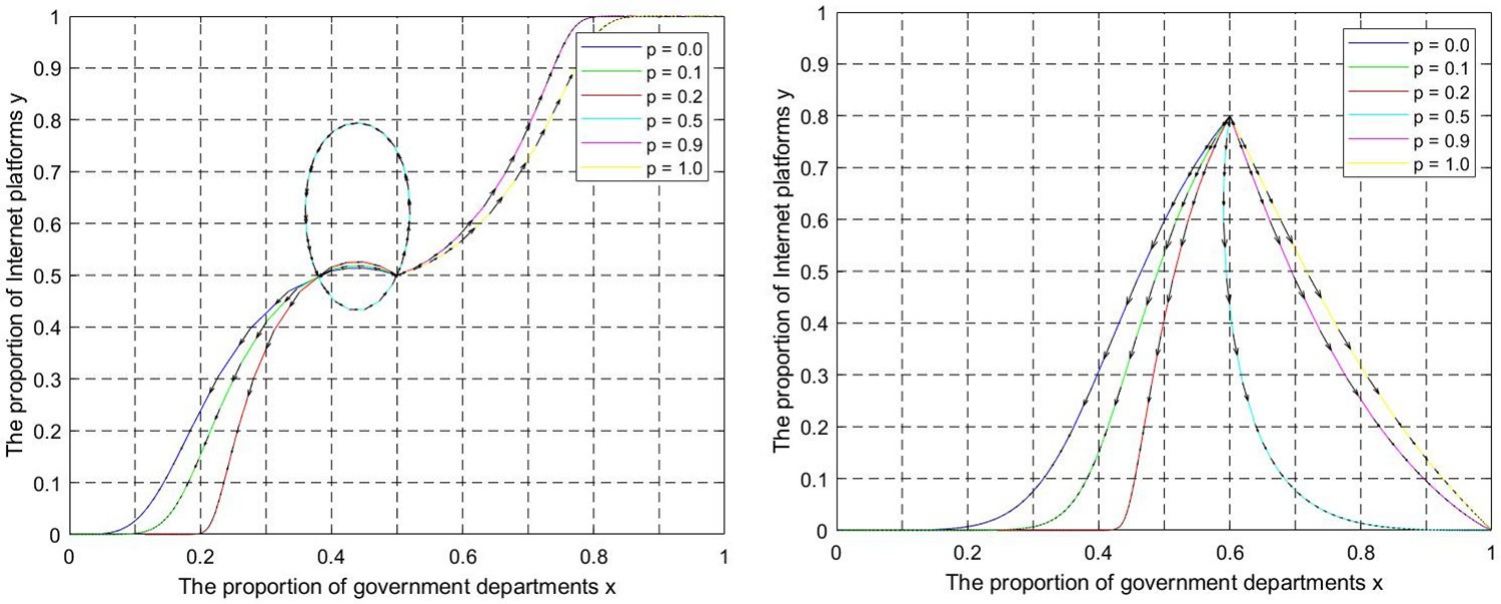
The influence of industry association participation on the ESS. (a) The punishment intensity z>z_0_. (b) The punishment intensity z<z_0_.

**Fig 11:**
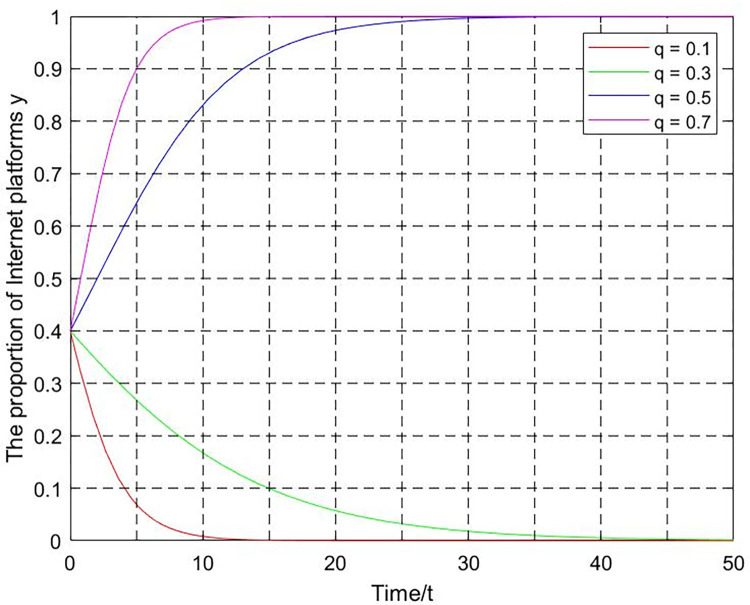
The influence of platform user participation on internet platforms’ strategies.

**Fig 12:**
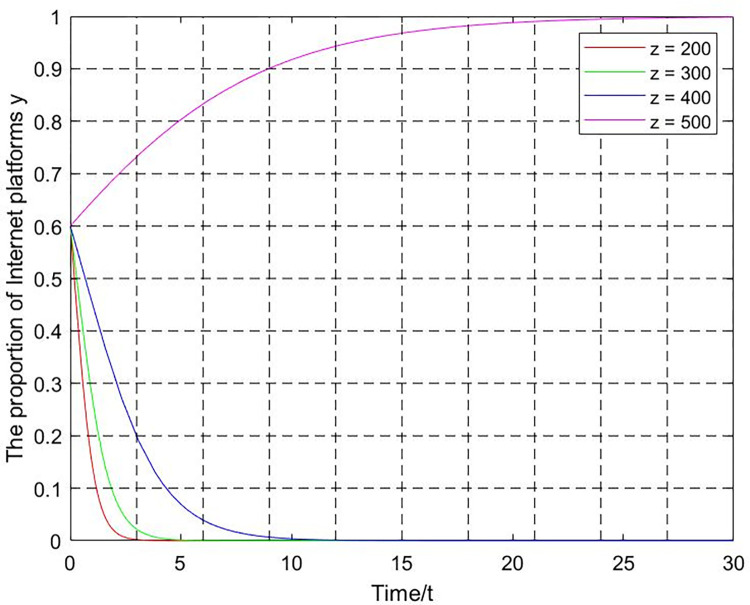
The influence of punishment on Internet platforms’ strategies.

**Fig 13:**
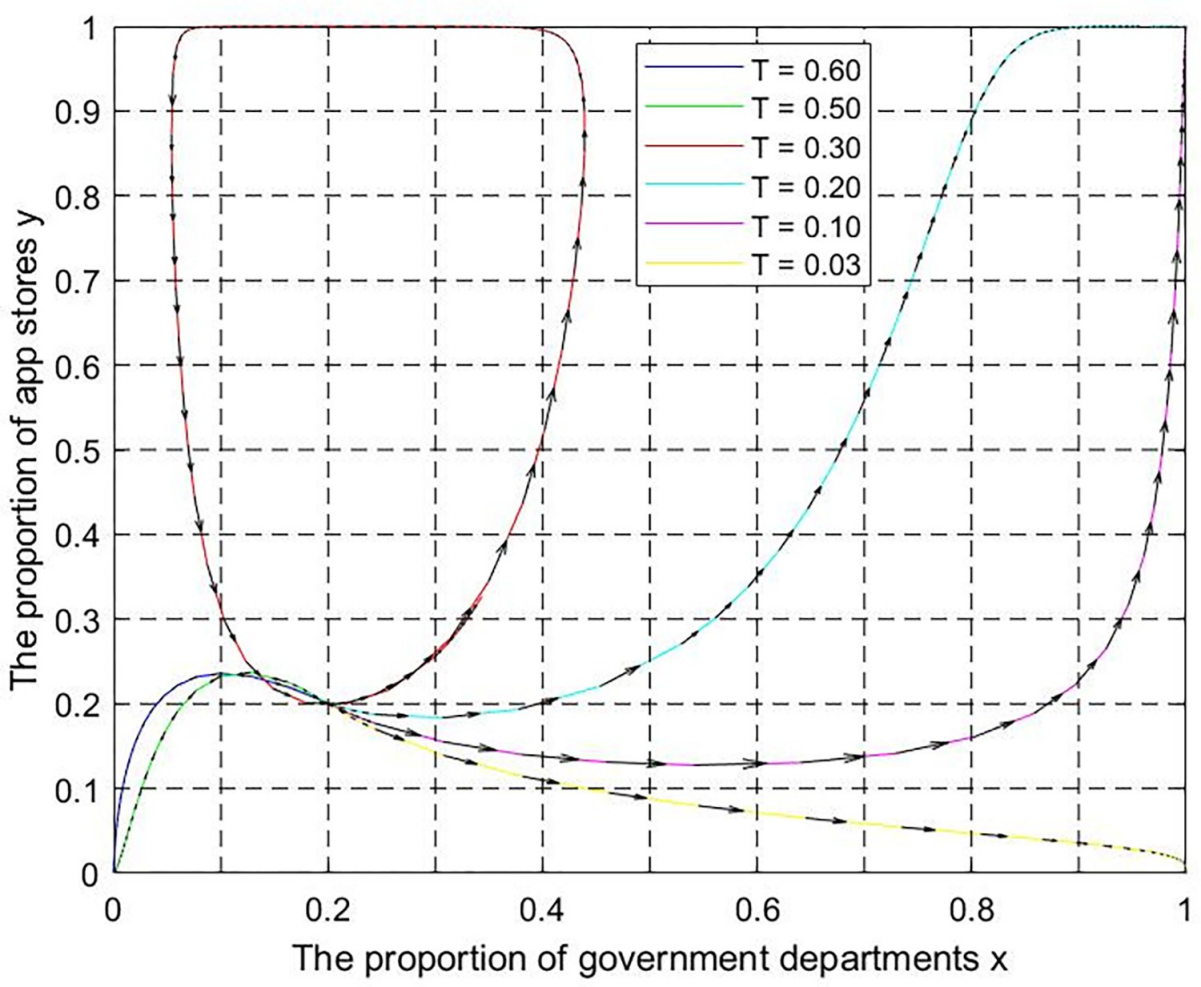
The influence of supervision intensity T on the ESS of the game system.

(1) Industry association participation p. Let T = 0.1, q = 0.1, m = 2, k = 0.001. The evolutionary trajectory of the game subject’s strategy is shown in Fig 10. According to the z_0_ expression, z_0_ = 180. When z>z_0_(z = 500), as industry association participation increases, the system’s ESS will skip state B and realize the transition from state A′→C′→D′. When z<z_0_(z = 5), as industry association participation increases, the system’s ESS will not reach C′ and D′ due to light punishment intensity and can only realize the transition from state A′→B′.

(2) Platform user participation q. Let x = 0.4, z = 5, T = 0.1, m = 2, k = 0.001, platform user participation q = 0.1,0.3,0.5,0.7. According to the replication dynamic Eq (14), the evolution curve of the fair competition ratio y as the value of q changes is shown in Fig 11. The Internet platform group’s strategy choice tends to be unfair competition when the platform user participation is low p = 0.1, 0.3. With the continuous increase of platform user participation until p = 0.5, the internet platform group’s strategy choice changes to fair competition. According to the replicator dynamic equation, the threshold value is p_0_ = 0.375. The internet platform strategy evolves toward fair competition when p>0.375. Conversely, The strategy evolves toward unfair competition when p<0.375.

(3) Punishment intensity z. Let x = 0.4,q = 0.5, T = 0.1, m = 2, k = 0.001, punishment intensity z = 200,300,400,500. The evolutionary curve of y changing with the z is shown in Fig 12. The strategy choice of the Internet platforms tends to be unfair competition when the punishment intensity is low (z = 200,300). The strategy choice of the Internet platforms tends to be fair competition as the punishment intensity increases until z = 400. According to the replication dynamic Eq (14), the threshold value is z = 475. The internet platform strategy choice evolves toward fair competition when z>475. Conversely, the strategy evolves toward unfair competition when z<475.

(4) Supervision intensity T. Let p = 0.5,q = 0.5, z = 300, m = 4, k = 0.01,T = 0.6,0.5,0.3,0.2,0.1,0.02. The evolutionary trajectory is shown in Fig 13. The governments choose the negative supervision strategy due to supervision cost constraints when the supervisory intensity is high (T = 0.6,0.5). The internet platforms adopt the unfair competition strategy without external supervision. With the continuous reduction of supervision intensity, the strategic choices of the two groups are interdependent when T = 0.3. With further reduced supervision intensity, the governments adopt the positive supervision strategy when T = 0.2,0.1. The Internet platforms adopt the fair competition strategy, and the system will evolve toward state D′. As the supervision intensity drops to an extremely low level when T = 0.03. Even if governments choose the positive supervision strategy, the internet platforms will still choose the unfair competition strategy, and the system will evolve toward state B′. Therefore, governments need to keep the supervision intensity within a reasonable range to guide the game system to evolve toward the ideal state.

According to Figs 10–13, if the increase in industry association participation, platform user participation, and punishment is limited, the evolution trajectory and evolution time will become shorter. However, it will still not be able to promote the transformation of the system from the negative state to the positive state. For government departments, it is necessary to grasp the degree of supervision based on factors such as supervision capabilities and supervision costs. Excessive supervision intensity will negatively affect the game system’s evolution to the positive state. In short, the evolutionary equilibrium point of the game system is the result of the joint action of multiple critical parameters. The game system can be guided to lock in the positive state by keeping critical parameters within a reasonable threshold range.

As mentioned above, Figs 6–9 illustrate the evolutionary paths of the game system when the critical parameters take values in various conditional intervals. The ESS in the figures is consistent with the equilibrium points A, B, C, and D in Table 3, and the equilibrium points A’, B’, C’, and D’ in Table 5, confirming propositions 5–8. Figs 10–13 depict the impact of increasing critical parameter values on the evolutionary paths of the game system. The evolutionary stable results in these figures align with propositions 9–11. Overall, the model simulation analysis provides adequate support for the construction, solution, and analysis of the evolutionary game model, validating the model’s scientific and rational nature.

## Model-to-reality matching analysis

According to the above, the evolutionary path of the game system can be described as (0,0)→(1,0)→(x_1_,y_1_)→(1,1). This section combines the reality of Chinese government’s UCIP supervision, divides the supervision process into four stages of “openness, inclusiveness, campaign-style, and normalization,” and matches it with the model evolution path.

(1) The "openness" supervision stage (before 2015). The platform economy is in its infancy and under the "Openness" supervision stage. Since 2008, China’s economic scale has slowed down and entered the "new normal" stage, and the economic development mode has gradually changed from traditional extensive to intensive. The internet platforms have grown wildly as emerging things due to the vast industry market and low barriers to entry. Various internet platforms such as e-commerce, social networking, and life entertainment have emerged in an endless stream, bringing significant changes to people’s shopping, social networking, and life entertainment. At the same time, various internet platforms are scrambling to seize market share and increase economic interests, and unfair competition incidents frequently occur. From the standpoint of the Chinese government, it chooses negative regulation due to the following three considerations. First, there needs to be more policy legislation, and the applicability of traditional industry supervisory policies in the field of platform economy is significantly reduced. The supervision method and how to define unfair competition still need to be determined. Second, the internet platform is a new thing. Its unfair competition is hidden and complicated. They need to make it easier to investigate and obtain evidence. The government has excellent disadvantages in data, technology, and human resources. It needs more experience and capabilities, resulting in high supervision costs. Third, emerging modes such as the platform economy are essential in promoting economic growth. Excessive administrative supervision may curb platform innovation, which is not conducive to the prosperity and development of the macroeconomy, and the supervision returns obtained are relatively small.
  At this stage, the Chinese government has not yet introduced relevant policies and regulations, and the traditional supervision policies do not apply to internet platforms, which are emerging things. They cannot effectively curb internet platform violations. Rather than solving the development problems of the platform economy through market mechanisms, more attention is paid to protecting the development of internet platforms rather than supervision.
(2) The "inclusiveness" supervision stage (2015–2018). The platform economy is in the growth stage and belongs to the "Inclusiveness" supervision stage. Since 2015, the Chinese government has followed the new round of the information technology revolution and the development trend of the global digital economy. It has successively introduced a series of macro policies such as "internet +," "information consumption," and "information economy," which have further promoted the development of the digital platform economy’s development and provided a strong impetus for China’s economic growth. On the one hand, governments have a deeper understanding of internet platforms’ operation and management. In addition, governments’ responsibilities have become more explicit. They have gradually improved and innovated the supervision methods, effectively improving supervision capabilities and reducing supervision costs. On the other hand, the Chinese government still adheres to the principle of "inclusive innovation and prudent supervision" to protect the emerging platform economy. Under this policy background, cross-border and peer competition among internet platforms is fierce as the platform economy grows rapidly. The internet platforms will continue to seize market share and increase operating profits through unfair competition. The supervisory policies from 2015 to 2018 are shown in Table 9.

**Table 9:** The Chinese government’s policies related to platform economy from 2015 to 2018.

Policy	Department	Time	Content
《"Guiding Opinions on Actively Promoting the "Internet Plus" Action"》	The State Council	July 2015	Accelerate the deep integration and innovative development of the internet and various fields; Relax market access restrictions for internet-integrated products and services, increase fiscal and taxation support, and improve financing services.
《"13th Five-Year Plan for the Development of National Strategic Emerging Industries"》	The State Council	December 2016	Tailor-made supervision modes for new business forms such as "internet +" and sharing economy that can be seen in terms of development prospects and potential risks; For uncertain emerging fields, strengthen monitoring and analysis, encourage inclusive development, and introduce supervisory measures that must be carefully studied and repeatedly demonstrated to avoid overly strict control.
《government working report》	The State Council	March 2017	Based on the principles of encouraging innovation, inclusiveness and prudence, formulate supervisory rules for emerging industries.

At this stage, the Chinese government adheres to the principle of inclusiveness to new industries, new formats, and new business modes such as the platform economy. Its macro policies still focus on promoting the development of the platform economy. This "inclusive supervision" method cannot effectively deter internet platform violations, and the platform economic supervision has entered the " Inclusiveness " supervision stage.

(3) The "campaign-style" supervision stage (2018-present). The platform economy is expanding, which belongs to the " Campaign-style " supervision stage. Since 2018, thanks to favorable macro policies and broad market demand, the scale of the platform economy has been overgrown. According to statistics from the China Academy of Information and Communications Technology, the market value of the platform economy reached $3.5 trillion in 2020, a year-on-year increase of 56.3%. With the accelerated expansion of the scale and number of internet platforms, unfair competition has intensified, and risks have accumulated and accumulated, causing massive damage to industry development and economic society. In this regard, the Chinese government has introduced a series of supervision policies, and relevant departments have launched specific rectification actions. These actions focus on addressing internet platforms that disrupt market order, infringe on user rights, threaten data security, violate resource and qualification management regulations, and exhibit other unfair competition behaviors. While the government encourages the development of the platform economy, it also increases supervision over its chaos. The supervisory policies after 2018 are shown in Table 10.

**Table 10:** The Chinese government’s policies related to platform economy after 2018.

Policy	Department	Time	Content
"Guiding Opinions on Facilitating the Standardized and Healthy Development of the Platform Economy"	The State Council	August 2019	Adhere to the "inclusive and prudent" supervisory principle and constantly innovate supervisory concepts and methods; Encourage the establishment of platforms for consumer complaints and rights protection; Encourage industry associations and other social organizations to introduce industry service norms and self-discipline conventions.
"Guidelines for Implementing Subject Responsibilities on internet Platforms"	The Market Supervision Bureau	October 2021	Do not engage in monopolistic behaviors such as monopoly agreements and abuse of market dominance; Do not use platform rules and data, algorithms, and other technical means to implement price discrimination, price gouging, low price dumping, and other unfair competitive behaviors.
"Several Opinions on Promoting the Standardized, Healthy and Sustainable Development of the Platform Economy"	The Development and Reform Commission	January 2022	Encourage industry associations to take the lead in formulating group standards and industry self-discipline conventions; Strengthen social supervision and explore a supervision mechanism involving the public and third-party organizations.
Revised version of Anti-Monopoly Law	The National People’s Congress	August 2022	Operators must not use data and algorithms, technology, capital advantages, and platform rules to engage in monopolistic behavior.

At this stage, the Chinese government still adheres to the supervision principle of "inclusiveness and prudence" and innovates supervisory methods. They impose strict penalties on violations with high potential risks and adverse severe consequences but have yet to formulate stringent punishment measures for general violations with minor consequences. At the same time, it is proposed to strengthen the role of industry associations, the public, and other entities in the governance of the platform economy and to build a supervision structure of pluralistic co-governance. Combined with the actual situation, China is in its current state.

(4) The "normalization" supervision stage (unknown). Through the Chinese government’s long-term exploration and practice, a modern governance system for the platform economy has been successfully built. The competition in the Internet platform market is orderly, and a healthy and benign development trend is maintained. The Chinese government regularly reviews the applicability of current supervision policies based on platform characteristics. They continue to improve the supervision mechanism of the full-process platform economy, encompassing pre-guidance, in-process regulation, and post-event supervision. Additionally, they aim to create a closed-loop supervisory ecology, incorporating legislation, law enforcement, and justice. The Chinese governments have deeply applied new technologies such as big data, blockchain, and artificial intelligence to supervise and enforce the platform economy. The supervision capacity has been continuously enhanced, and the efficiency of law enforcement has been dramatically improved. Under the "normalization" supervision of the government, the unfair competition behavior of Internet platforms will be effectively deterred through an effective punishment and restraint mechanism. This will lead to a significant reduction in unfair competition behavior on Internet platforms. Finally, industry associations continuously strengthen industry self-discipline, cooperating with supervisory authorities to carry out standardized governance and purifying the competitive environment of internet platforms. At the same time, platform users’ awareness of rights protection has been greatly enhanced. Information on UCIP has been transmitted to the government through unimpeded reporting and complaint channels. As a result, the expected benefits of unfair competition strategies have been significantly reduced by individuals or entities "leave the platform." Thanks to the efforts of governments, industry associations, and platform users, UCIP has been effectively regulated, ensuring the healthy and orderly development of the platform economy market.

At this stage, The Chinese government has developed a complete platform economy supervision concept, system, mode, and ecology at this stage. This comprehensive framework effectively safeguards market fairness and justice, contributing to the construction of a healthy and standardized platform economy market order. The platform economy has become the backbone of China’s high-quality economic development.

## Conclusions, implications and limitations

### Conclusions

Based on the actual situation in China, this paper uses evolutionary game theory to explore the effectiveness of the "multi-agent co-governance" mode in solving the UCIP problems. The main conclusions are as follows:

1. Under the " multi-agent co-governance " game model, ESS is affected by multiple factors. Industry association participation, power space, platform user participation, information sharing level, and punishment intensity have a positive effect on ESS. The effect of supervisory intensity on ESS is uncertain, but an optimal supervisory intensity can be found to maximize the probability of ESS evolving to (1,1).
2. As the parameter value conditions of industry association participation p and platform user participation q (or punishment intensity z) change, the system presents three evolutionary stable strategies and a periodic random state. The evolutionary path can be described as (0,0)→(1,0)→(x_1_,y_1_)→(1,1).
3. Currently, China is in the (x_1_,y_1_) stage. The game system will be guided from the state (x_1_,y_1_) to (1,1) transition by increasing the industry association and platform user participation.
4. The system has no ESS (0,1) in both modes. That is, government administrative intervention is the essential condition for regulating UCIP. It is challenging to achieve self-discipline of competitive behavior on internet platforms by relying solely on market mechanisms.

### Implications and limitations

Based on the research findings above, the following management insights can be drawn for the Chinese government to design a reasonable UCIP regulation model. (1) Governments should leverage emerging technologies such as big data and blockchain to innovate supervision methods and tools. It is essential to balance supervisory intensity, avoiding situations where regulation intensity becomes excessively high or too lax. (2) Improve the collaborative participation mechanism for industry associations and platform users, issue specific norms and rules, and increase their enthusiasm for participation. (3) Develop reasonable penalty mechanisms based on internet platforms’ scale and the severity of violations. These mechanisms constrain UCIP behavior while avoiding excessive penalties that could stifle industry development. (4) The government should continuously revise and improve policies and regulations in real time to avoid issues with no legal basis due to the rapid iteration of internet platform business models and market dynamics.

Indeed, this study has certain limitations that require further optimization and improvement by future scholars. First, in addition to industry associations and platform users, the news media, the general public, and other social organizations play crucial roles in UCIP. The research did not consider the roles of these entities in the "multi-agent co-governance" supervisory system. Second, this paper introduced industry associations and platform users as critical parameters in the game model to explore their impact on the evolution of the game system. This simplification of model construction and analysis may reduce the accuracy and validity of the research process and results. Third, this paper employed a general, traditional MATLAB simulation approach and qualitative model-to-reality matching analysis to validate the accuracy of the model analysis process and results. However, the model verification still needs to be achieved through empirical research. Fourth, the research was based on the specific conditions in China to construct an evolutionary game model for UCIP supervision, providing theoretical references for adequate UCIP supervision by the Chinese government. However, its applicability in other countries and regions remains to be verified.

In light of the above limitations, the following directions for future research are suggested. First, a more comprehensive analysis of the stakeholders involved in UCIP supervision should be conducted. The game model should be optimized and refined, and the impact of the actions of all parties on the effectiveness of UCIP supervision should be analyzed. This may yield more valuable research findings. Second, a real-world data set should be gathered for the model parameters. The model should be validated through empirical research. Finally, construct tailor evolutionary game models to the specific circumstances of UCIP regulation in different countries and regions.

## Supporting information

S1 File (DOCX)
