# Supplementary material for: Research on the "multi-agent co-governance" system of unfair competition on internet platforms: Based on the perspective of evolutionary game
Source: PLoS One. 2024 Apr 18;19(4):e0301627. doi: 10.1371/journal.pone.0301627 (PMC11025923; doi:10.1371/journal.pone.0301627)
Supplement: S1 File — (DOCX) [file pone.0301627.s001.docx]

**Equilibrium point (0,0)**

dxdt = @(t, x) [x(1)*(1-x(1))*(-3-2*x(2)); (-5+2.05*x(1))*x(2)*(1-x(2))];

tspan = [0 10];

x1range = [0 1];

x2range = [0 1];

initialPoints = [0.2 0.4; 0.5 0.5; 0.8 0.6; 0.9 0.9; 0.3 0.9; 0.9 0.3; 0.6 0.8];

figure;

hold on;

for i = 1:size(initialPoints, 1)

x0 = initialPoints(i, :)';

[t, sol] = ode45(dxdt, tspan, x0);

plot(sol(:, 1), sol(:, 2));

quiver(sol(1:end-1, 1), sol(1:end-1, 2), diff(sol(:, 1)), diff(sol(:, 2)), 0.5, 'Color', 'b');

end

numGrids = 10;

gridStep = 1 / numGrids;

for i = 1:numGrids-1

line([i * gridStep, i * gridStep], [0, 1], 'Color', 'k', 'LineStyle', '--');

end

for i = 1:numGrids-1

line([0, 1], [i * gridStep, i * gridStep], 'Color', 'k', 'LineStyle', '--');

end

xlim(x1range);

ylim(x2range);

ax = gca;

ax.Box = 'on';

ax.XAxis.Color = 'k';

ax.YAxis.Color = 'k';

xlabel('The proportion of government departments x');

ylabel('The proportion of Internet platforms y');

scatter(initialPoints(:, 1), initialPoints(:, 2), 'filled', 'MarkerFaceColor', 'red');

dxdt = @(t, x) [x(1)*(1-x(1))*(-2.7-2.2*x(2)); (-5+2.25*x(1))*x(2)*(1-x(2))];

tspan = [0 10];

x1range = [0 1];

x2range = [0 1];

initialPoints = [0.2 0.4; 0.5 0.5; 0.8 0.6; 0.9 0.9; 0.3 0.9; 0.9 0.3; 0.6 0.8];

figure;

hold on;

for i = 1:size(initialPoints, 1)

x0 = initialPoints(i, :)';

[t, sol] = ode45(dxdt, tspan, x0);

plot(sol(:, 1), sol(:, 2));

quiver(sol(1:end-1, 1), sol(1:end-1, 2), diff(sol(:, 1)), diff(sol(:, 2)), 0.5, 'Color', 'b');

end

numGrids = 10;

gridStep = 1 / numGrids;

for i = 1:numGrids-1

line([i * gridStep, i * gridStep], [0, 1], 'Color', 'k', 'LineStyle', '--');

end

for i = 1:numGrids-1

line([0, 1], [i * gridStep, i * gridStep], 'Color', 'k', 'LineStyle', '--');

end

xlim(x1range);

ylim(x2range);

ax = gca;

ax.Box = 'on';

ax.XAxis.Color = 'k';

ax.YAxis.Color = 'k';

xlabel('The proportion of government departments x');

ylabel('The proportion of Internet platforms y');

scatter(initialPoints(:, 1), initialPoints(:, 2), 'filled', 'MarkerFaceColor', 'red');
